# Severe Acute Respiratory Syndrome Coronavirus-2 Lambda Variant Collected from a Child from Arkansas and Sequenced

**DOI:** 10.1128/mra.00007-23

**Published:** 2023-02-13

**Authors:** Lori Wong, Christopher Randolph, Emily Kanwischer, Ashton Ingold, Bobby L. Boyanton, Jay Taylor, Rachel A. Frenner, Susan E. Harley, Jordan T. Bird, Timothy J. Thurman, Sangam Kandel, David W. Ussery, Stephanie D. Byrum, Darrell Dinwiddie, Daryl Domman, Uday K. Chalwadi, Joshua Kennedy

**Affiliations:** a Department of Biomedical Informatics, University of Arkansas for Medical Sciences, Little Rock, Arkansas, USA; b Department of Biochemistry and Molecular Biology, University of Arkansas for Medical Sciences, Little Rock, Arkansas, USA; c Department of Pathology, University of Arkansas for Medical Sciences, Little Rock, Arkansas, USA; d Department of Pathology, Arkansas Children’s Hospital, Little Rock, Arkansas, USA; e Arkansas Children’s Research Institute, Little Rock, Arkansas, USA; f Department of Pediatrics, University of New Mexico, Albuquerque, New Mexico, USA; g Center for Global Health, Dept of Internal Medicine, University of New Mexico Health Sciences Center, Albuquerque, New Mexico, USA; h Department of Pediatrics, University of Arkansas for Medical Sciences, Little Rock, Arkansas, USA; i Department of Internal Medicine, University of Arkansas for Medical Sciences, Little Rock, Arkansas, USA; Queens College Department of Biology

## Abstract

An eleven-year-old tested positive for SARS-CoV-2 Lambda variant. Sequencing was performed on the Oxford Nanopore and the Illumina NextSeq 500. Both platforms identified all 7 of the synonymous mutations in the sample, while all 28 nonsynonymous mutations were identified from Oxford Nanopore and 20 nonsynonymous mutations were identified from Illumina.

## ANNOUNCEMENT

An 11-year-old white female with a history of frequent urinary tract infections and methicillin-resistant Staphylococcus aureus skin abscesses tested positive for the severe acute respiratory syndrome coronavirus-2 (SARS-CoV-2; Coronaviridae: *Betacoronavirus*) Lambda variant (Pango lineage C.37) in central Arkansas in the summer of 2021. The patient was asymptomatic at the time of testing; however, she had been exposed to a caregiver who was symptomatic. She was not vaccinated against SARS-CoV-2, as she did not qualify due to age.

A single nasopharyngeal swab was collected and placed into 3 mL M4RT transport medium (Remel, San Diego, CA). The sample was tested for SARS-CoV-2 using the Aptima SARS-CoV-2 (Panther System, Hologic, San Diego, CA) nucleic acid amplification assay.

This work was completed under University of Arkansas for Medical Sciences (UAMS) institutional review board (IRB) no. 273461. Sequencing of human clinical specimens at University of New Mexico, Health Sciences Center (UNM HSC) was completed under the IRB-approved study Human Research Review Committee (HRRC) no. 19-592.

RNA extraction was performed on a thawed aliquot from the residual nasopharyngeal swab sample using the MagMax viral/pathogen nucleic isolation kit (Applied Biosystems) on the Kingfisher Flex automated instrument (Thermofisher).

Viral RNA was reverse transcribed to generate cDNA as described (1). cDNA was amplified by PCR using the ARTIC-v.3 primer set; libraries were generated using the ligation sequencing (SQK-LSK109) and native barcoding expansion 96 (EXP-NBD196) kits from Oxford Nanopore Technologies. Sequencing was done following nCoV-2019 sequencing protocol v3 (LoCost) V.3 (1) using a Minion R9.4.1 flow cell on an Oxford Nanopore GridION device with the MinKNOW application, resulting in 320× coverage of the genome with 27,993 down-sampled reads. Base-calling of Nanopore raw reads (FAST5 format) and trimming sequencing adaptors were performed using Guppy 3.4.5 (2). Demultiplexed FASTQ files were subsequently processed using the ARTIC Network Bioinformatics pipeline (3). Sequencing reads were quality filtered using the gupplyplex script, and reference-based genome assembly was done using the nanopolish minion script from the ARTIC pipeline. The consensus sequence generated a length of 29,752 bp and mapped to NC_045512.2 as the reference.

**FIG 1:**
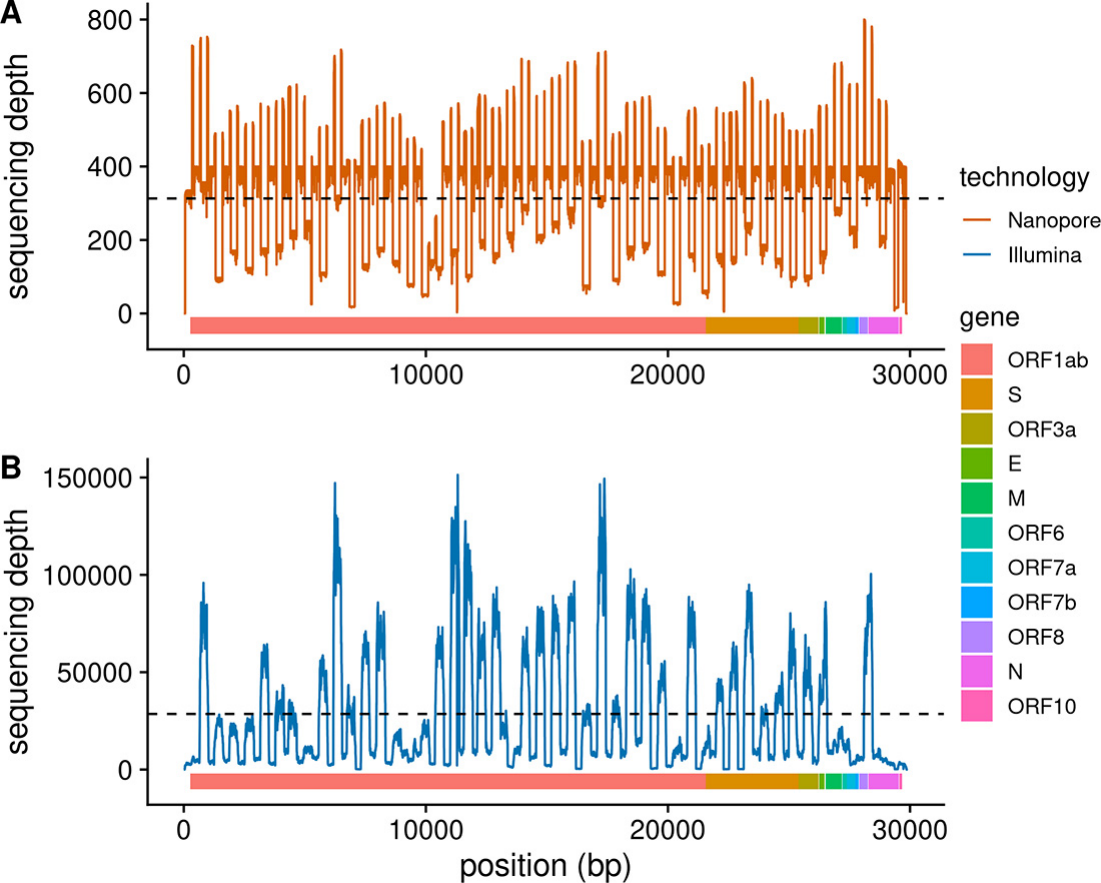
Sequencing depth for the V00303 patient sample. Sequencing read depth is shown across the SARS-CoV-2 genome. The genes are identified by the colored bar. (A and B) Oxford Nanopore (A) and Illumina COVIDSeq test (B) sequencing platforms are shown.

Libraries were prepared using the Illumina COVIDseq test kits and were sequenced on a NextSeq 500 instrument with Artic-v.3 primers, resulting in 11,640,339 sequencing reads with 28,500× coverage (Fig. 1). FASTQ files were generated for each sample after demultiplexing the raw sequencing data on Base Space Sequence Hub. FASTQ files were concatenated across lanes and then processed with fastp v0.23.2 (4) to remove adaptors, trim low-quality bases, and merge overlapping paired-end reads. Reads were mapped to the NC_045512.2 sequence using the MEM algorithm of BWA v0.7.17-r1188 (5) with default options, and genome assembly was done using iVar v1.3.1 (6), generating a consensus sequence of 29,864 base pairs.

Nextclade v1.10.2 (7) with default parameters was used to characterize mutations and amino acid changes in the consensus sequence generated from both Nanopore and Illumina sequencing and identified all 7 of the synonymous mutations in the sample from both sequencing platforms. All 28 nonsynonymous mutations were identified from Oxford Nanopore sequencing, while 20 nonsynonymous mutations were identified from Illumina sequencing (Table 1). Overall, there is strong evidence suggesting the patient had a positive identification of the SARS-CoV-2 Lambda variant using both Oxford Nanopore and Illumina sequencing platforms.

**TABLE 1:** Defining lambda variant amino acid mutations^*a*^

Gene	Defining mutation	Amino acid position	Reference amino acid	Lambda amino acid	Nanopore sample amino acid^*b*^	Illumina sample amino acid^*b*^
S	G75V	75	G	V	V	V
S	T76I	76	T	I	I	I
S	R246-	246	R			Low coverage
S	S247-	247	S			Low coverage
S	Y248-	248	Y			Low coverage
S	L249-	249	L			Low coverage
S	T250-	250	T			Low coverage
S	P251-	251	P			Low coverage
S	G252-	252	G			Low coverage
S	D253N	253	D	N	N	Low coverage
S	L452Q	452	L	Q	Q	Q
S	F490S	490	F	S	S	S
S	D614G	614	D	G	G	G
S	T859N	859	T	N	N	N
ORF1a	T1246I	1246	T	I	I	I
ORF1a	P2287S	2287	P	S	S	S
ORF1a	F2387V	2387	F	V	V	V
ORF1a	L3201P	3201	L	P	P	P
ORF1a	T3255I	3255	T	I	I	I
ORF1a	G3278S	3278	G	S	S	S
ORF1a	S3675-	3675	S			
ORF1a	G3676-	3676	G			
ORF1a	F3677-	3677	F			
ORF1b	P314L	314	P	L	L	L
N	P13L	13	P	L	L	L
N	R203K	203	R	K	K	K
N	G204R	204	G	R	R	R
N	G214C	214	G	C	C	C

a The lambda variant has 28 amino acid mutations relative to the SARS-CoV-2 refence sequence. Mutations are named according to the reference amino acid, the amino acid position in the protein, and the variant amino acid.

b The sample amino acid columns show the amino acid found in the patient sample.

### Data availability.

The consensus sequence for the Illumina platform has been deposited to GenBank (OP589812); raw sequences were deposited into the NCBI SRA database (SRR21817417). The Oxford Nanopore assembly sequence has been deposited to GenBank (OM462008); raw sequences were deposited into the NCBI SRA database (SRR22753266).
